# A Refractory Case of Sydenham Chorea Managed With Intravenous Pulse-Dose Methylprednisolone

**DOI:** 10.1155/crpe/3533017

**Published:** 2025-10-23

**Authors:** Andrea Weitz, Inna Kaminecki, Clark Azubuike, Alexandria L. Rivas, Sai Pranathi Bingi, Jennifer E. Wilson, Jessyca T. Cripps, Michael Mitchell, Mary Baiyeri, Maria Gasque

**Affiliations:** ^1^Department of Emergency Medicine, Texas Tech University Health Sciences Center, Lubbock, Texas, USA; ^2^Texas Tech University Health Sciences Center School of Medicine, Lubbock, Texas, USA; ^3^Department of Pediatrics, Texas Tech University Health Sciences Center, Lubbock, Texas, USA; ^4^Pediatric Neurology, Texas Tech University Health Sciences Center, Lubbock, Texas, USA

## Abstract

We describe the case of an eight-year-old female presenting with abrupt-onset involuntary movements, emotional lability, and gait disturbances, consistent with Sydenham's chorea (SC). Her condition deteriorated despite initial antibiotic treatment and symptomatic management. Notable findings included elevated antistreptolysin O titers and antideoxyribonuclease B antibodies, suggestive of recent Group *A Streptococcus* infection. Brain magnetic resonance imaging revealed punctate gliosis in the supratentorial white matter, and extensive workup excluded alternative diagnoses. Despite escalating therapy with valproic acid, clonidine, and haloperidol, the patient exhibited persistent choreiform movements and emotional dysregulation. High-dose corticosteroids (methylprednisolone) were initiated, resulting in significant symptomatic improvement and restoration of ambulatory function. Long-term prophylaxis with benzathine penicillin G was implemented to prevent recurrence. SC remains an under-researched complication of acute rheumatic fever, with treatment often extrapolated from limited case reports and expert consensus. This case underscores the potential role of corticosteroids in refractory SC. This case also highlights the complexity of managing prolonged SC and the importance of individualized, multifaceted treatment strategies.

## 1. Introduction

Acute rheumatic fever (ARF) is an immune-mediated consequence of Group *A Streptococcus* (GAS) pharyngitis. The diagnosis of ARF is made using revised Jones criteria [1, 2]. The diagnosis of ARF for low-risk populations is based on the presence of 2 major criteria or 1 major and 2 minor criteria. Major manifestations include carditis, arthritis, chorea, subcutaneous nodules, and erythema marginatum. Minor manifestations include polyarthralgia, fever ≥  38.5C, erythrocyte sedimentation rate (ESR)  ≥  60 mm in the first hour and/or C-reactive protein (CRP) ≥  3 mg/dL, and prolonged PR interval.

Confirmation of prior GAS pharyngitis is also required for diagnosis. This includes evidence of elevated or rising antistreptolysin O (ASO) titers or antideoxyribonuclease B (anti-DNase B) antibodies, a positive throat culture for GAS, or a positive rapid GAS test in a child presenting with clinical signs of streptococcal pharyngitis [3].

Sydenham's chorea (SC) typically manifests several months after the onset of GAS pharyngitis and may present as an isolated finding. For this reason, the presence of SC alone is sufficient to diagnose ARF [2]. Chorea is a relatively rare manifestation of ARF, with its incidence varying widely across different regions of the world [4]. Studies indicate that 10% to 40% of patients with ARF develop chorea [4, 5]. SC usually appears 6 to 8 weeks after a GAS pharyngitis infection and is not associated with skin infections. It is more common in females than males (3:1 ratio) and predominantly affects children aged 5–18, with a peak incidence at 8-9 years old [4].

The treatment of SC focuses on symptom management and preventing recurrence. All patients with SC should initially receive intramuscular benzathine penicillin as part of ARF management [6]. Symptom management for SC varies based on severity. While most cases resolve spontaneously, patients with moderate to severe or prolonged symptoms may require additional treatment. However, the optimal approach to treating chorea remains unclear, as it has not been extensively studied.

There is no universally established definition for refractory SC. However, some literature describes refractory SC as cases where symptoms are severe and/or do not respond to standard symptomatic treatments, such as neuroleptics or antiepileptic drugs. When conventional therapy fails, immunomodulatory options, such as corticosteroids, intravenous immunoglobulin (IVIG), or plasmapheresis, may be considered [7–9].

## 2. Case Details

We report the case of an eight-year-old female who presented with abnormal movements of the upper and lower extremities, accompanied by emotional lability that began approximately 4 days prior. The family also noted the appearance of an erythematous rash on her bilateral arms and inner thighs. Her mother reported that the patient initially exhibited heightened emotional incongruence, which was followed by the development of abnormal movements described as “fidgeting.” The patient also complained of an unusual sensation in her mouth, described as “feeling hair in her mouth,” which caused her to grind her teeth.

Initially, the patient was taken to an outside emergency department, where she was diagnosed with GAS pharyngitis after a positive strep test. She received an intramuscular dose of ceftriaxone and was started on amoxicillin. However, after discharge, her abnormal movements worsened, progressing to vigorous jerking. Due to the severity of the movements, she was unable to dress herself or walk. She also experienced bowel and urinary incontinence, prompting her referral to our emergency department for further evaluation.

A review of her outside medical records revealed that imaging (noncontrast computed tomography of the head) and laboratory tests (complete blood count, comprehensive metabolic panel, and urine drug screen) were all within normal limits.

On examination, the patient appeared restless. Her temperature was 97.4°F (36.3°C), heart rate 116 beats per minute, respiratory rate 22 breaths per minute, blood pressure 97/58 mm Hg, and oxygen saturation 99% on room air. She was alert, with warm, dry skin and no evidence of a rash. Her pupils were equal and reactive to light, with normal extraocular movements. No pharyngeal erythema or exudates were observed.

The chest examination revealed mild tachycardia with a regular rhythm, no cardiac murmur, and clear breath sounds bilaterally. The abdominal examination showed a soft, nondistended abdomen without evidence of organomegaly. Neurologically, she exhibited no focal deficits but demonstrated an unsteady gait while ambulating. She also displayed abnormal flailing movements of the upper and lower extremities, facial grimacing, and involuntary tongue movements. When asked to suppress these movements, she was able to do so only briefly, for a few seconds.

Further review of the patient's history revealed that she had experienced four episodes of streptococcal pharyngitis within the past year, the most recent occurring 4 months ago. Each diagnosed episode was treated with a 10-day course of amoxicillin.

Laboratory evaluation showed a white blood cell count of 8.8 K/μL (8.8 × 10^9^/L), a platelet count of 356 K/μL (356 × 10^9^/L), and a hemoglobin level of 14 g/dL (140 g/L). Inflammatory markers included CRP level of 0.3 mg/dL (28.5 nmol/L) and ESR of 21 mm/hr. ASO titers were significantly elevated at 1748 IU/mL (reference range: < 150 IU/mL), and anti-DNase B levels were elevated at 444 units/mL (reference range: < 376 units/mL).

Urinalysis and thyroid studies were normal, with a thyroid-stimulating hormone level of 0.81 mcIU/mL (reference range: 0.5–5 mcIU/mL) and a free T4 level of 1.57 ng/dL (reference range: 0.8–2.8 ng/dL). A complete metabolic panel also yielded normal results. Electrocardiography and chest X-ray findings were unremarkable.

The differential diagnosis was broad and included ARF with SC, anti-N-methyl-D-aspartate (NMDA) receptor encephalitis, seizure disorder, systemic lupus erythematosus, other causes of encephalitis, acute disseminated encephalomyelitis, toxic ingestion, Wilson's disease, and familial chorea.

While in the emergency department, the patient received a dose of haloperidol, which resulted in the complete resolution of her involuntary movements for approximately 2 h. She also received a dose of midazolam, but it had no effect on her symptoms.

Following admission, the patient continued to exhibit involuntary movements, including repetitive opening and closing of her mouth and constant flailing of the upper and lower extremities. She demonstrated spooning of her hands (wrist flexion with metacarpophalangeal hyperextension), which worsened with upper extremity movements. The patient was unable to walk independently and required support. Her speech was staggered due to persistent involuntary tongue movements. She could not form full sentences and was only able to yell out isolated words. Emotional lability was also observed. However, no sensory deficits were noted.

Additional laboratory evaluation showed negative antinuclear antibodies and normal complement studies with C3 complement level of 106 mg/dL (reference range: 90–180 mg/dL) and C4 complement level of 10 mg/dL (reference range: 10–40 mg/dL). Throat culture was negative for beta-hemolytic *Streptococcus*. The direct antiglobulin test was negative.

ARF was our primary suspected diagnosis, so an echocardiogram was performed, which revealed no cardiac pathology. The electrocardiogram was also normal for the patient's age. The differential diagnosis included anti-NMDA receptor encephalitis. A pelvic ultrasound yielded normal results, and the patient's serum beta-HCG level was negative. In addition, serum NMDA receptor antibody testing was negative.

**Figure 1:**
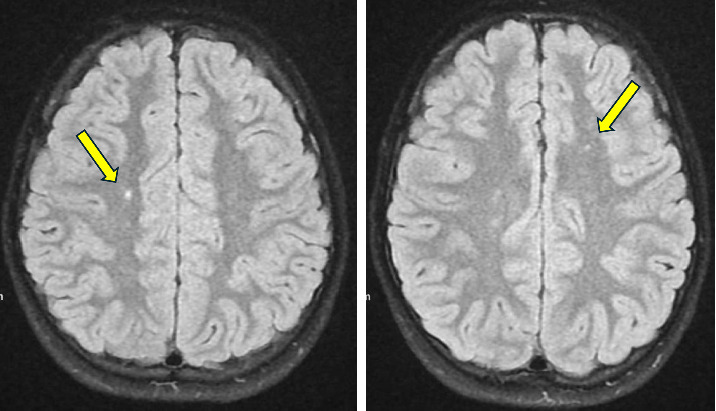
Areas of gliosis within the supratentorial white matter.

The patient was evaluated by an infectious disease specialist, who recommended secondary prophylaxis for ARF with benzathine penicillin G, 1.2 million units intramuscularly every 3–4 weeks. She was also assessed by a pediatric neurologist. Due to suspected SC, the patient received a loading dose of valproic acid (20 mg/kg), followed by a maintenance dose of 10 mg/kg twice daily. In addition, clonidine 0.1 mg was initiated at bedtime. Magnetic resonance imaging (MRI) of the brain revealed punctate areas of presumed gliosis within the supratentorial white matter (Figure 1). Cerebrospinal fluid (CSF) studies showed a normal white blood cell count of 7/mm^3^, red blood cells of 7/mm^3^, protein of 24 mg/dL, and glucose of 52 mg/dL. The meningitis and encephalitis panel by polymerase chain reaction was negative, as were NMDA receptor antibodies in the CSF. CSF culture yielded negative results, and no oligoclonal bands were identified. The patient's presentation and evaluation were consistent with ARF with SC.

During the patient's hospitalization, her choreiform movements continued to worsen, prompting an increase in the clonidine dose to 0.15 mg. Despite this adjustment, the patient experienced persistent fluctuations in her symptoms. She received haloperidol at night as needed, resulting in short periods of symptom improvement. By Day 4 of hospitalization, there were still no significant improvements in her symptoms. The decision was made to initiate high-dose steroid therapy, starting with methylprednisolone 1 g/day for 3 days. The valproic acid dose was also increased to 15 mg/kg twice daily. Following steroid administration, the patient demonstrated noticeable improvements in her involuntary movements, regaining the ability to walk without assistance. She was able to speak in full sentences, smile, and rest with minimal symptoms, though her symptoms worsened with movement or in crowded environments, likely due to anxiety. After completing 3 days of high-dose steroids, a prednisone taper over 30 days was initiated. By Day 8 of hospitalization, her involuntary movements had improved, though she remained emotionally labile and required lorazepam as needed. Her anxiety, possibly exacerbated by the high-dose steroids, may have contributed to intermittent worsening of her involuntary movements. To address these issues, the patient was started on low-dose haloperidol (0.5 mg at night) and low-dose clonazepam (0.5 mg twice daily). At that time, her medication regimen included valproic acid 20 mg/kg twice daily, haloperidol 1.5 mg at night for three nights, clonidine 0.1 mg at night, clonazepam 0.5 mg twice daily for 3 days, then once daily for 3 days, and lorazepam as needed.

During the patient's hospitalization, she was evaluated by a child psychologist, who assisted her in developing coping skills to manage and regulate her emotions. In addition, the patient began physical, occupational, and speech therapy during her hospital stay.

The patient began to show clinical improvement by Day 11 of hospitalization, with decreased involuntary movements. She started ambulating with assistance but continued to use a wheelchair. She was able to hold conversations using short sentences and remained on a steroid taper. On Day 14, she was discharged to an inpatient rehabilitation facility.

The patient followed up with an infectious disease specialist and a pediatric neurologist. She continued secondary prophylaxis with penicillin G benzathine, receiving 1.2 million units intramuscularly. After completing her rehabilitation course, she reported feeling much better. However, she still experiences mild chorea symptoms, noticeable only during moments of frustration, 9 months after her initial diagnosis. Her current medications include valproic acid at 20 mg/kg twice daily and clonidine 0.1 mg at bedtime. She will continue penicillin G benzathine prophylaxis every 28 days until she turns 21 years old. The patient is scheduled to follow up with the neurology service in 3 months, with consideration to discontinue valproic acid at her next visit.

## 3. Discussion

SC is most commonly diagnosed in females aged 5–15 years and presents with involuntary movements of the upper and lower extremities, facial grimacing, gait abnormalities, and difficulties with writing and speaking. It can also present as “chorea paralytica,” characterized by generalized weakness and hypotonia [10, 11]. Neuropsychiatric symptoms are also common, including emotional lability, irritability, tics, and obsessive-compulsive features [12, 13]. Diagnosing SC is challenging, primarily due to the difficulty in identifying early symptoms and the delayed onset of manifestations. Caregivers may misinterpret the restless movements and involuntary facial expressions of SC as behavioral issues, such as hyperactivity, clumsiness, or intentional lack of cooperation. Symptoms of SC typically appear between 1 and 8 months after the inciting infection [14], often leading to delays in both diagnosis and treatment.

The pathogenesis of SC is not yet fully understood. However, molecular mimicry is believed to play a major role in its development. This process involves antibodies directed against GAS antigens that cross-react with self-antigens, resulting in a dysregulated immune response [15]. A variety of GAS antigens are implicated in this mechanism. In SC, it is proposed that antibasal ganglia antibodies target specific regions of the basal ganglia in the brain, leading to elevated dopamine release and subsequent movement disorders [16, 17].

The diagnosis of SC is primarily clinical and is often made in patients with other findings consistent with ARF. There is no specific laboratory test for SC. Current data on MRI findings associated with SC are limited. MRI is typically performed to rule out other causes of chorea, as there are no definitive imaging findings specific to SC. However, MRI findings may include signal hyperintensities in the white matter, brainstem, and basal ganglia, particularly in the caudate nucleus [10, 12, 18, 19]. In one study, cranial MRI abnormalities were identified in 47% of patients with SC [10]. Other studies have shown that, in most cases, MRI changes resolve within 4–6 months, though some patients may still exhibit changes after 12 months of follow-up [10, 12]. In our patient, punctate areas of gliosis were observed in the supratentorial white matter. Although this finding is nonspecific, it can occur in patients with SC.

As the pathogenesis of SC involves dopamine receptor antibodies, commonly used medications include dopamine antagonists such as haloperidol, pimozide, and phenothiazines [7, 20]. Another frequently used group of medications is antiepileptics, including valproic acid, carbamazepine, diazepam, and levetiracetam [7, 20, 21]. A recent systematic review revealed that the most frequently used medications in SC were haloperidol and valproic acid [6]. However, the available studies are highly scattered, making it difficult to draw firm conclusions about the best management approach for SC. Current literature does not strongly support one treatment modality over another. One study comparing carbamazepine, haloperidol, and valproic acid found that valproic acid was more effective in treating SC. It was associated with faster symptom resolution and fewer side effects compared to haloperidol [20]. The use of benzodiazepines in SC has shown unclear treatment outcomes, and data on their efficacy in SC remain very limited [6, 7].

Literature also supports the use of IVIG, corticosteroids, and plasmapheresis in some cases, as the disease has an autoimmune origin [6, 22–24]. These modalities are usually reserved for patients with severe chorea or those who have not responded to other treatments. Reports in the literature describe the use of oral prednisone, intravenous methylprednisolone, and corticotropin [9, 23–25]. Studies have shown that patients treated with steroids alone or in combination with other medications experienced faster symptom resolution.

The amount and quality of studies on the use of IVIG in SC are limited. IVIG treatment should be reserved for patients who have not responded to other modalities or who present with severe conditions such as “chorea paralytica” [7].

A randomized controlled trial compared IVIG, plasmapheresis, and corticosteroids in the treatment of SC [22]. There were no significant differences in outcomes between the groups; however, clinical improvement was more rapid in patients who received IVIG or plasma exchange compared to those in the prednisone group.

One case report documented dramatic improvement after five rounds of plasmapheresis in a severe case of “chorea paralytica,” where antiepileptics, neuroleptics, and corticosteroids had failed [8].

Our case represents a patient with prolonged symptoms that were refractory to a variety of medications. Improvement was observed after corticosteroids were initiated. However, by that time, she was already on multiple other medications, and the improvement could have been due to the combined effects of these treatments.

Prevention of further recurrence of SC is a crucial element of the treatment plan. Long-term antibiotic therapy with penicillin helps minimize the risk of rheumatic heart disease [26]. After treatment, most patients with SC typically achieve full recovery within approximately 1–7 months. However, some authors have reported that even 2 years after the diagnosis of SC, 50% of patients still experience symptoms of chorea [9].

## 4. Conclusion

We present a case report of SC that was refractory to multiple medications. The patient showed improvement after the administration of high-dose corticosteroids. To date, the management of SC is not well studied, and the optimal treatment approach remains unknown. Our patient was also started on clonidine, a treatment not previously described, which warrants further investigation in the management of SC.

## References

[1] Dajani A. S. (1992). Guidelines for the Diagnosis of Rheumatic Fever: Jones Criteria, 1992 Update. *JAMA*.

[2] Gewitz M. H., Baltimore R. S., Tani L. Y. (2015). American Heart Association Committee on Rheumatic Fever, Endocarditis, and Kawasaki Disease of the Council on Cardiovascular Disease in the Young. Revision of the Jones Criteria for the Diagnosis of Acute Rheumatic Fever in the Era of Doppler Echocardiography: A Scientific Statement From the American Heart Association. *Circulation*.

[3] Gerber M. A., Baltimore R. S., Eaton C. B. (2009). Prevention of Rheumatic Fever and Diagnosis and Treatment of Acute Streptococcal Pharyngitis: A Scientific Statement from the American Heart Association Rheumatic Fever, Endocarditis, and Kawasaki disease Committee of the Council on Cardiovascular Disease in the Young, the Interdisciplinary Council on Functional Genomics and Translational Biology, and the Interdisciplinary Council on Quality of Care and Outcomes Research: Endorsed by the American Academy of Pediatrics. *Circulation*.

[4] Beier K., Lui F., Pratt D. P. (2024). Sydenham Chorea. *StatPearls [Internet]. Treasure Island (FL)*.

[5] Eshel G., Lahat E., Azizi E., Gross B., Aladjem M. (1993). Chorea as a Manifestation of Rheumatic Fever--a 30-Year Survey (1960–1990). *European Journal of Pediatrics*.

[6] Tariq S., Niaz F., Waseem S. (2023). Managing and Treating Sydenham Chorea: A Systematic Review. *Brain and Behavior*.

[7] Dean S. L., Singer H. S. (2017). Treatment of Sydenham’s Chorea: A Review of the Current Evidence. *Tremor and Other Hyperkinetic Movements*.

[8] Miranda M., Walker R. H., Saez D., Renner V. (2015). Severe Sydenham’s Chorea (Chorea Paralytica) Successfully Treated With Plasmapheresis. *Journal of Clinical Movement Disorder*.

[9] Eyre M., Thomas T., Ferrarin E. (2024). Treatments and Outcomes Among Patients With Sydenham Chorea: A Meta-Analysis. *JAMA Network Open*.

[10] Ekici A., Yakut A., Yimenicioglu S., Bora Carman K., Saylısoy S. (2014). Clinical and Neuroimaging Findings of Sydenham’s Chorea. *Iranian Journal of Pediatrics (English edition)*.

[11] Miyake C. Y., Gauvreau K., Tani L. Y., Sundel R. P., Newburger J. W. (2007). Characteristics of Children Discharged From Hospitals in the United States in 2000 With the Diagnosis of Acute Rheumatic Fever. *Pediatrics*.

[12] Faustino P. C., Terreri M. T., da Rocha A. J., Zappitelli M. C., Lederman H. M., Hilário M. O. E. (2003). Clinical, Laboratory, Psychiatric and Magnetic Resonance Findings in Patients With Sydenham Chorea. *Neuroradiology*.

[13] Punukollu M., Mushet N., Linney M., Hennessy C., Morton M. (2016). Neuropsychiatric Manifestations of Sydenham’s chorea: A Systematic Review. *Developmental Medicine and Child Neurology*.

[14] Gilbert D. L. (2023). Sydenham Chorea. National Organization for Rare Disorders (NORD).

[15] Church A. J., Cardoso F., Dale R. C., Lees A. J., Thompson E. J., Giovannoni G. (2002). Anti-Basal Ganglia Antibodies in Acute and Persistent Sydenham’s chorea. *Neurology*.

[16] Singer H. S., Loiselle C. R., Lee O., Garvey M. A., Grus F. H. (2003). Anti-Basal Ganglia Antibody Abnormalities in Sydenham Chorea. *Journal of Neuroimmunology*.

[17] Kirvan C. A., Swedo S. E., Kurahara D., Cunningham M. W. (2006). Streptococcal Mimicry and Antibody-Mediated Cell Signaling in the Pathogenesis of Sydenham’s Chorea. *Autoimmunity*.

[18] Emery E. S., Vieco P. T. (1997). Sydenham Chorea: Magnetic Resonance Imaging Reveals Permanent Basal Ganglia Injury. *Neurology*.

[19] Robertson W. C., Smith C. D. (2002). Sydenham’s Chorea in the Age of MRI: A Case Report and Review. *Pediatric Neurology*.

[20] Peña J., Mora E., Cardozo J., Molina O., Montiel C. (2002). Comparison of the Efficacy of Carbamazepine, Haloperidol and Valproic Acid in the Treatment of Children With Sydenham’s Chorea: Clinical Follow-Up of 18 Patients. *Arquivos de Neuro-Psiquiatria*.

[21] Genel F., Arslanoglu S., Uran N., Saylan B. (2002). Sydenham’s chorea: Clinical Findings and Comparison of the Efficacies of Sodium Valproate and Carbamazepine Regimens. *Brain and Development*.

[22] Garvey M. A., Snider L. A., Leitman S. F., Werden R., Swedo S. E. (2005). Treatment of Sydenham’s Chorea With Intravenous Immunoglobulin, Plasma Exchange, or Prednisone. *Journal of Child Neurology*.

[23] Barash J., Margalith D., Matitiau A. (2005). Corticosteroid Treatment in Patients With Sydenham’s Chorea. *Pediatric Neurology*.

[24] Cappellari A. M., Rogani G., Filocamo G., Petaccia A. (2023). Corticosteroid Treatment in Sydenham Chorea: A 27-Year Tertiary Referral Center Experience. *Children*.

[25] Paz J. A., Silva C. A., Marques-Dias M. J. (2006). Randomized Double-Blind Study With Prednisone in Sydenham’s Chorea. *Pediatric Neurology*.

[26] Cardoso F., Vargas A. P., Oliveira L. D., Guerra A. A., Amaral S. V. (1999). Persistent Sydenham’s Chorea. *Movement Disorders*.

